# Stakeholders’ perspectives on the implementation of school hearing screening in Botswana

**DOI:** 10.4102/jphia.v16i1.1239

**Published:** 2025-05-09

**Authors:** Meshack Moepeng, Shajila Singh, Lebogang Ramma

**Affiliations:** 1 Department of Health and Rehabilitation Sciences, Division of Communication Sciences and Disorders, Faculty of Health Sciences, University of Cape Town, Cape Town, South Africa; 2 Department of Audiology, Princess Marina Hospital, Gaborone, Botswana

**Keywords:** low- and middle-income countries, perspectives, policy implementation, policy knowledge, school health policy, school hearing screening

## Abstract

**Background:**

School-based hearing screening programmes play an important role in identifying and providing appropriate intervention services to children with progressive, late-onset, or acquired hearing loss.

**Aim:**

To describe the knowledge and perspectives of government stakeholders within the Ministry of Health and the Ministry of Education and Skills Development on the implementation of school entry hearing screening programmes in Botswana.

**Setting:**

The study was conducted in two administrative districts: Gaborone and the South-East District, Botswana.

**Methods:**

A qualitative descriptive design was used. Fifteen key government stakeholders, including policymakers and service providers (audiologists, nurses, Grade 1 teachers), were purposefully sampled. Semi-structured interviews were conducted with each stakeholder. All audio recordings were transcribed verbatim. Reflexive thematic analysis was performed with the assistance of NVivo 12 software.

**Results:**

There was variable knowledge of policy with most of the service providers not being aware of the existing current national school health policy. All stakeholders interviewed demonstrated universal support for implementation of the policy in relation to school-based hearing screening. Some service providers suggested that hearing screening services could be integrated into existing school health programmes. Barriers and facilitators for policy implementation were also identified.

**Conclusion:**

Stakeholders’ knowledge and support of the school health policy suggest that there is potential for the implementation of school-based hearing screening programmes.

**Contribution:**

This study created awareness of a provision for school hearing screening in an existing policy that had not been implemented. The findings present an opportunity for advocating for the implementation of school hearing screening programmes.

## Introduction

Hearing loss is a major barrier to effective learning and contributes to poor literacy rates among primary school learners.^1,2^ The implementation of school hearing screening programmes could reduce the burden of hearing loss on learners’ academic performance, social-emotional maturity, and cognitive development.^3,4^ In countries where early hearing detection and intervention (EHDI) programmes are well established and universal newborn hearing screening programmes are routinely implemented, school hearing screening programmes target late-onset hearing loss and identification of hearing loss in children who, for various reasons, may have missed being screened during the neonatal period.^5,6^

In low- and middle-income countries, where EHDI programmes largely do not exist,^7,8^ school entry hearing screening programmes may be the earliest point of access for identifying and providing appropriate intervention to school-age children with progressive, late-onset, acquired or undetected hearing loss.^9,10,11^ The implementation of school hearing screening programmes remains fragmented globally due to the lack of political and financial commitment from the relevant stakeholders and a lack of specific national school hearing screening policy frameworks to support the implementation of such programmes.^11^

In Botswana, like many African countries, there are no EHDI programmes,^7^ and there are very few audiologists in the health sector to establish such programmes. However, the existence of a national school health policy,^12^ and the potential to have easy access to a large number of children in one place^9^ provide a practical opportunity to start school hearing screening programmes in the country. The effective provision of school hearing screening services requires appropriate policies and guidelines to ensure that there is consistency in the implementation and operation of the hearing screening programmes among the involved stakeholders. Perspectives and attitudes of stakeholders may reflect the barriers, facilitators, and key drivers necessary for policy implementation.^13^ Stakeholders’ opinions and expectations have the potential to influence programme funding and implementation.^14^ Exploring the views of key stakeholders may provide valuable insights that could inform policy formulation and implementation^13^ and be utilised when advocating for the implementation of school hearing screening programmes.

This study aimed to describe the knowledge and perspectives of relevant government stakeholders within the Ministry of Health (MoH) and the Ministry of Education and Skills Development (MoESD) on the implementation of school entry hearing screening programmes in Botswana.

## Research methods and design

### Study design

A qualitative descriptive design was employed in this study. This design utilised semi-structured interviews^15^ to explore the relevant government stakeholders’ knowledge and perspectives regarding the implementation of school entry hearing screening programmes in Botswana.

### Study population

There were two categories of participants from the education and health sectors. The first category comprised policymakers from MoH and MoESD in Gaborone. The second category consisted of frontline service providers within Gaborone and the South-East administrative districts.

#### Inclusion criteria

- Policymakers: Government officials involved in policy making and knowledgeable on school health service provision.
- Frontline service providers:
  ▪ Health personnel: Audiologists and nurses involved in school health services, working in public hospitals or clinics.
  ▪ Education personnel: Grade 1 public school teachers.

All participants were required to be fluent in either English or Setswana, as these are the two official languages in Botswana. The participants were also required to have at least 6 months’ work experience in their current position to ensure that they have adequate insights about their role to enable them to provide rich information required for this study.

#### Recruitment and enrolment

Permission was obtained from all the relevant authorities: (1) MoESD and the MoH headquarters in Gaborone, Botswana, (2) health facilities (two clinics and two hospitals) in Gaborone and the South-East administrative districts, and (3) primary schools in the South-East district. The first author contacted all potential participants at MoESD and the MoH headquarters, health facilities, and schools via telephone to recruit and enrol them in the study.

### Sampling method

Purposeful sampling was used to select information-rich participants.^16,17^ To achieve maximum variation, the sample included health and education personnel, consisting of policymakers and frontline service providers (teachers, nurses, and audiologists) working in rural, peri-urban, and urban areas^16,17^ from two administrative districts (Gaborone and the South-East district).

### Sample size

Sample size was determined to be sufficient when data saturation was reached and there were no new themes emerging from the data.^18,19,20,21^ In this study, data saturation (i.e., sufficient sample size) was reached after interviewing a total of 15 participants from the MoH and the MoESD.

### Description of participants

The 15 key government stakeholders who participated in this study comprised audiologists (*n* = 3), nurses (*n* = 5), Grade 1 teachers (*n* = 4), and policymakers (*n* = 3). Of the 15 participants, 73% (*n* = 11) were females, and 27% (*n* = 4) were males (Table 1). All three policymakers (an advisor at the MoH, a principal education officer, and a regional education director at the MoESD) were males, while the frontline service providers (audiologists, nurses, and teachers) comprised 11 females and 1 male. The age of the participants ranged from 30 years to 57 years, with a median age of 39 years. The policymakers tended to be older (median age = 56 years; range = 52–57 years) relative to the frontline service providers (median age = 37 years; range = 30–54 years) (Table 1). The duration in their current job roles ranged from 2 to 33 years, with a median duration of 6 years. The median duration in the current job role for policymakers was 6 years (range = 2–11 years). The median duration in the current job role for the frontline service providers was 8 years (range = 3–33 years).

**TABLE 1:** Biographical profile of the interviewed participants.

Participants	Sex	Age (years)	Location	Duration in the current job role (years)
**Ministry of Health**				
Advisor	Male	56	Urban	2
Nurse 1	Female	39	Peri-urban	14
Nurse 2	Female	39	Peri-urban	15
Nurse 3	Female	34	Rural	6
Nurse 4	Female	42	Peri-urban	10
Nurse 5	Female	38	Urban	3
Audiologist 1	Male	30	Urban	5
Audiologist 2	Female	36	Peri-urban	10
Audiologist 3	Female	31	Urban	5
**Ministry of Education and Skills Development**				
Principal Education Officer	Male	52	Urban	6
Regional Education Director	Male	57	Urban	11
Teacher 1	Female	33	Rural	5
Teacher 2	Female	33	Peri-urban	3
Teacher 3	Female	54	Rural	33
Teacher 4	Female	53	Peri-urban	23

### Pilot interviews

Prior to beginning data collection, pilot interviews were conducted to (1) practise interviewing techniques, (2) identify and reformulate any unclear questions, and (3) determine the time taken to complete each interview. Two key stakeholders from the South-East district, a school health nurse from a peri-urban area and a Grade 1 teacher from a rural area, were selected to participate. The results of the pilot study suggested that no changes needed to be made to the interview guide or questions. The pilot interviews further indicated that each interview would take about 30 min – 45 min, which was accordingly reflected in the participant information letter. The data obtained from the two stakeholders who participated in the pilot interviews were included in the main study data analysis for the following reasons: (1) the two stakeholders provided detailed interview responses that contributed to some of the emerging themes, and (2) no changes were made to the interview guide and to the way the interviews were conducted.^22,23^

### Data collection tools

#### Interview guide

The questions in the interview guide were structured to be open-ended, to explore the participants’ knowledge about the topic areas and to allow them to express their views freely, with detailed responses to reveal important ideas and emerging themes.^24^ Follow-up probe questions were used to obtain details and to clarify information provided during the interview.^25,26,27^ The questions used in the interview guide, based on the reviewed literature, were formulated to guide the authors on the focus areas to be covered during the interview and to elicit in-depth responses from the participants.^28^

Questions focused on determining the participants’ knowledge of existing policies on school hearing screening, views regarding accessibility and utilisation of such policies, support for policy implementation, and allocation of resources for implementing school hearing screening programmes in Botswana. Questions were also framed to solicit perspectives on integrating school entry hearing screening programmes into existing school health programmes. Questions for frontline service providers (i.e., audiologists, nurses, and teachers) were also formulated to probe perspectives on the current status of school health and hearing screening programmes, and the level of support for the implementation of such programmes within their specific education region or District Health Management Team (DHMT) area. A question specific to teachers or nurses sought their views on upskilling members of their professions to include hearing screening within their existing work roles.

#### Interview platform

Interviews were conducted virtually^29^ using the Microsoft Teams application, and all interviews were audio recorded for transcription and analysis later.

### Data collection procedures

All the participants enrolled in this study were scheduled for an interview. Prior to the scheduled interview, participants were emailed an information letter, a consent form, a biographical information form, and the interview questions. Access to questions prior to the interview afforded participants the opportunity to prepare for in-depth responses ahead of time,^30,31^ allowing for the collection of rich and comprehensive data.^32^ A few days before the interview, the first author contacted each participant by telephone to review the purpose of the study and answer any questions about the study. The participants emailed back written informed consent and biographical information.

The first author, who is fluent in English and Setswana, conducted a semi-structured virtual interview with each participant in the language preferred by the participant (English or Setswana). However, 14 interviews were conducted primarily in English, and 1 interview was conducted in Setswana. After each interview, the audio recordings were saved in encrypted and password-protected files in a computer not linked to any network. The audio recordings of each participant were transcribed verbatim and then uploaded to NVivo 12 software^33^ for analysis.

### Data analysis

Reflexive thematic analysis was performed on the transcribed data, which was organised and managed using NVivo 12 software.^26^ The following six steps for conducting a reflexive thematic analysis, developed by Braun and Clarke^34^ were followed: (1) data familiarisation: each interview transcript was read and re-read to immerse and familiarise the researcher with the data content, (2) data coding: phrases that identified aspects of data that might answer the research question from each interview transcript were collated and grouped into codes within the NVivo 12 software, (3) searching for themes: patterns of shared meaning within the created codes were identified and combined to generate themes, (4) reviewing themes: the initial themes were reviewed and modified as necessary to ensure that they accurately represent the transcribed data, (5) defining and naming themes: each theme presented in the final analysis was named and defined to provide its meaning in relation to specific aspects of the data it captures, and (6) writing up the report: the analysed data was written up in a research report.

### Trustworthiness and rigour

To address trustworthiness and rigour, the current study adopted Lincoln and Guba’s^35^ criteria for trustworthiness: credibility, transferability, dependability, and confirmability. Credibility, as suggested by Ghafouri and Ofoghi^36^ was established by collecting data from a wide range of key informants, consisting of different professionals (advisors, audiologists, directors, nurses, and teachers) and sites from two ministries (MoESD and MoH), which presented data from diverse viewpoints and experiences within the relevant context. Member checking^37^ was achieved by sending interview transcripts to participants to enable them to evaluate the transcripts, correct errors, and provide additional information where necessary. Transferability was established by providing thick descriptions of data collected from the participants during the interviews.^36,37^ Dependability was established through in-depth methodological description, outlining all the research processes from data collection to data analysis, to ensure that the study is replicable.^37^ Confirmability was ensured by using an audit trail; documenting and describing all the research steps taken from the study conceptualisation to data analysis and reporting of findings.^35^

### Ethical considerations

Ethics approval for this study was granted by the University of Cape Town, Faculty of Health Sciences, Human Research Ethics Committee (HREC REF: 728/2020) and the MoH (Botswana), Health Research and Development Division (REF: HPDME 13/18/1). The study adhered to the ethical principles of the Declaration of Helsinki.^38^

## Results

This study revealed the following five overarching themes: variable knowledge of policy existence, universal support for policy implementation, barriers to policy implementation, facilitators of policy implementation, and implementing school hearing screening services (Table 2).

**TABLE 2:** Themes and sub-themes generated from the stakeholders’ transcripts.

Main themes	Sub-themes
1. Variable knowledge of policy existence	1.1 Partial knowledge of policy among policymakers
	1.2 Lack of policy knowledge among service providers
2. Universal support for policy implementation	2.1 Resource mobilisation support
	2.2 Frontline service provision support
3. Barriers to policy implementation	3.1 Lack of collaboration in government departments
	3.2 Inadequate human and financial resources
	3.3 Lack of monitoring and evaluation
4. Facilitators of policy implementation	4.1 Improving policy dissemination and awareness
	4.2 Strengthening stakeholder collaboration in government departments
	4.3 Training of school and health personnel
5. Implementing school hearing screening services	5.1 Integration into existing school health programmes

### Theme 1: Variable knowledge of policy existence

#### Sub-theme 1.1: Partial knowledge of policy among policymakers

All the policymakers at the MoH and MoESD were aware of the existence of the current national school health policy, but only one of them (advisor at the MoH headquarters) was aware that the policy includes school hearing screening:

‘Yes, there is a school health policy which has produced a manual that guides how screening, including hearing screening in primary schools should be conducted.’ (Advisor, MoH)

‘I’m aware of the fact that our schools do have what is known as a school health policy … There are no policies for screening of hearing problems. We only come to be aware of a child having some hearing problems through observations from teachers who teach these learners and reports from parents …’ (Principal Education Officer, MoESD)

#### Sub-theme 1.2: Lack of policy knowledge among service providers

The majority (*n* = 9) of the service providers at the policy implementation level in both the education and health sectors (teachers, nurses, and audiologists) were not aware of the existence of any policy related to school hearing screening:

‘Currently there is no school health policy that I’m aware of … There is no school hearing screening policy that I know about …’ (Nurse 3, MoH)

‘As a teacher, I never came across any policy or guidelines document on school hearing screening.’ (Teacher 1, MoESD)

A few (*n* = 3) service providers from both the health and education sectors were aware of the existence of the national school health policy, but only one, a nurse from a peri-urban district hospital, was aware that school hearing screening is included within the national school health policy:

‘I only know of the school health policy of 1999 for primary schools … This policy involves the health of the environment, physical health including hearing screening, immunisation of children which is prevention of diseases.’ (Nurse 1, MoH)

All the stakeholders from the MoH and MoESD, including policymakers (advisor, principal education officer, and regional education director) and policy implementers (nurses, audiologists, and teachers), indicated that school hearing screening programmes in primary schools are currently non-existent. Two nurses contended that the policy implementation gap could be attributed to poor access to and a lack of awareness of the policy on school hearing screening among the policy implementers:

‘Our school health policy is not accessible … Most of our school health personnel have limited knowledge on the existing policy, and this may be one of the reasons why hearing screening is not conducted in school children.’ (Nurse 1, MoH)

‘In our district hearing screening has been omitted within the school health programmes, I think this omission could be caused by the fact that as nurses we do not have access to the relevant policy guiding school health services, and it is possible that most of us [*nurses*] do not know that hearing screening should be part of the school health programmes.’ (Nurse 3, MoH)

### Theme 2: Universal support for policy implementation

A key finding was that all the stakeholders, including policymakers, teachers, audiologists, and school health nurses, generally expressed willingness to support and personally participate in the implementation of school entry hearing screening policies or programmes in Botswana.

#### Sub-theme 2.1: Resource mobilisation support

The policymakers at the MoH and MoESD headquarters indicated that they would support the programme implementation processes by making human and financial resources available to the service providers:

‘My support for the implementation of the school entry hearing screening programmes will be huge … It is important that whatever resources are available we should pull them together. Uh, staffing resources, and even time should be provided for such.’ (Advisor, MoH)

‘I will definitely support that [*school hearing screening policy implementation*] … if we can have teachers being trained on how they can do it [*hearing screening*] we can definitely support that.’ (Principal Education Officer, MoESD)

#### Sub-theme 2.2: Frontline service provision support

The audiologists and nurses also indicated a willingness to be part of the service providers rolling out the school hearing screening programmes at any DHMT within the country. The teachers revealed that they would participate in the implementation process by accommodating the relevant personnel conducting hearing screening within their class schedule:

‘I will fully support the implementation of such a policy … if it requires me to be part of the entourage which will roll out the programme to our district and other district health management teams, I will be more than happy to take part in such.’ (Nurse 2, MoH)

‘I will also support the implementation of such a policy by accommodating the health personnel doing the hearing screening in my class schedule.’ (Teacher 1, MoESD)

### Theme 3: Barriers to policy implementation

#### Sub-theme 3.1: Lack of collaboration in government departments

The MoH stakeholders indicated that a lack of collaboration within and among the government departments negatively impacted the implementation of school hearing screening in accordance with the current school health policy:

‘One of the issues that we are dealing with is operation in silos. This should be a whole government approach, even though I said that this school health programme is driven by the three tripartite ministries [*Ministry of Health, Ministry of Education and Skills Development, and Ministry of Local Government and Rural Development*].’ (Advisor, MoH)

#### Sub-theme 3.2: Inadequate human and financial resources

All the stakeholders from the MoH and the MoESD reported that the lack of human and financial resources was a major barrier to policy implementation. The policymakers and the service providers from the health and education sectors highlighted that the scarcity of skilled personnel is a hindrance to the successful implementation of school hearing screening programmes:

‘The one major challenge is personnel. Lack of human resources, because if we have just two audiologists in the district, it is not possible for them to conduct hearing screening in so many schools in a year.’ (Nurse 1, MoH)

Stakeholders from the education and health sectors at all levels (policy making and policy-implementation level) were not aware of any funding structures or budget plans available for the provision of school hearing screening services. They underscored that lack of funding would hinder the provision of school hearing screening services in primary schools:

‘The number one challenge obviously is going to be resources, financial resources. One, there will be a need to mobilise training of more people to do the screening. That calls for money. If it [*hearing screening programme*] has to have devices that can be used, it will need money for that.’ (Principal Education Officer, MoESD)

#### Sub-theme 3.3: Lack of monitoring and evaluation

Four stakeholders – two nurses, a teacher, and a principal education officer – believed that a lack of monitoring and evaluation could be a hindrance to the implementation of school hearing screening programmes in Botswana:

‘We continue to omit a lot of things such as hearing screening when providing school health programmes due to lack of monitoring and evaluation systems to make appropriate follow-ups and recommendations on the progress of the implementation of such programmes.’ (Nurse 3, MoH)

### Theme 4: Facilitators of policy implementation

#### Sub-theme 4.1: Improving policy dissemination and awareness

Stakeholders at the policy implementation level, such as nurses and teachers, reiterated that making the policy accessible to those who will be implementing it will empower them and create a platform for initiating hearing screening programmes in primary schools:

‘I believe we should be made aware of the existing policies. If they are there, they should be made available in the DHMTs [*District Health Management Teams*] and in the clinics. That is, the policies should be placed where we can easily access them as nurses in the clinics.’ (Nurse 5, MoH)

A policymaker (advisor) and service providers (nurses and audiologists) at the MoH underscored the importance of raising awareness about school hearing screening to educate relevant stakeholders (the community, healthcare workers, teachers, and children) about the existing policy:

‘If we have a policy, let’s say about hearing screening, all stakeholders should be aware of it. … you need to educate the community, including the community of healthcare workers, teachers, parents, and children.’ (Nurse 1, MoH)

#### Sub-theme 4.2: Strengthening stakeholder collaboration in government departments

The MoH stakeholders highlighted that strengthening stakeholder collaboration within and between government departments may improve implementation of the existing school health policy:

‘This should be a whole government approach, even though I said that this school health programme is driven by the three tripartite ministries. There are other entities at other ministries and other departments who should be taking part as well. … our entities have to work together to ensure that we deliver as one, a package that can ensure that all the learners benefit from the programme. So, it is important that we all collaborate in this role.’ (Advisor, MoH)

#### Sub-theme 4.3: Training of school and health personnel

The stakeholders from both the health and education sectors suggested that the implementation of school hearing screening programmes will need appropriately trained hearing screening personnel. They were of the view that relevant frontline service providers such as audiology assistants, community health workers, nurses, and teachers could be trained to conduct hearing screening to enable them to effectively participate in the school hearing screening service provision. The nurses and teachers showed willingness for the members of their professions to be trained. They suggested that, after training, school hearing screening could be integrated into their existing work roles within the school health programmes:

‘I think it is going to be important for them [*nurses*] to be trained so that when they go to conduct school health screening, they incorporate hearing screening procedures as part of the multidisciplinary tasks they are doing.’ (Nurse 2, MoH)

‘Training teachers in school hearing screening will be a good, and very important step, because it will help the teachers to have the skills to identify learners with hearing problems.’ (Teacher 4, MoESD)

An audiologist from the MoH was of the view that audiology assistants and healthcare professionals with appropriate training could be utilised as school hearing screening personnel:

‘In my opinion, considering the shortage of audiologists in Botswana, I think any trained audiology assistant or healthcare professional who has undergone a training specifically for hearing screening can help to conduct school hearing screening.’ (Audiologist 2, MoH)

An advisor from the MoH suggested that community health workers could be considered as hearing screening personnel, as they are readily available within the communities in different districts:

‘There is the starting or the commencement of the community health workers programme, who will be working in the community and leveraging on the community structures that we have. A similar approach can be followed for providing school hearing screening services.’ (Advisor, MoH)

### Theme 5: Implementing school hearing screening services

#### Sub-theme 5.1: Integration into existing school health programmes

Most frontline service providers and policymakers from the MoH and MoESD acknowledged that school health programmes exist, particularly in the South-East Education region primary schools. Some nurses were of the view that school hearing screening services could be integrated into existing school health programmes to reduce learning interruptions when providing such services:

‘If we can take advantage of the available school health programmes to incorporate hearing screening services into such programmes, I think it will really help in our district as now the health screening activities will be collaborated, and it will reduce the amount of time that students will miss school or the learning process for this exercise to take place.’ (Nurse 2, MoH)

‘We conduct school health screenings every year, however, hearing screening is not included in our school health programmes. We need to include hearing screening in the school health programmes to identify learners with hearing problems and refer them on time for treatment.’ (Nurse 4, MoH)

## Discussion

The current study sought to determine the knowledge and perspectives of key stakeholders concerning school entry hearing screening programmes in Botswana. Knowledge about policy existence varied among the key government stakeholders from the MoH and the MoESD. The majority of the service providers from both the health and education sectors were not aware of the existence of the national school health policy. Some policymakers at national government departments were aware of the existence of the current national school health policy, but only one had knowledge about the provision in the policy pertaining to the implementation of school hearing screening. This limited knowledge among policymakers was surprising, as one of their key responsibilities is policy formulation, monitoring, and evaluation oversight.^39,40^ Shaibu and Phaladze^41^ in Botswana also reported limited knowledge of the existing school health policy among the key stakeholders, such as teachers, parents, and the community, and linked this lack of policy knowledge to low policy implementation.

The findings of this study reflect no implementation of the school hearing screening component of the national school health policy. This policy implementation gap could be attributed to inadequate access and lack of awareness of policy existence among the MoH and MoESD service providers.^39,42^ There is also no legislation associated with the current policy to make school hearing screening mandatory. In low- and middle-income countries, inadequate access to policies, lack of information relating to hearing screening services, and lack of relevant legislation have also been reported as major barriers to policy implementation and specifically to implementation of hearing screening programmes for children.^9,43,44,45,46^

Policymakers and service providers at the MoH underscored the importance of raising awareness about school hearing screening to educate relevant stakeholders such as the community, parents, healthcare workers, school administrators, and teachers about the existing policy. Adequate policy knowledge among key stakeholders can improve its acceptance and implementation uptake.^42,47,48^ The knowledge about the current national school health policy could be improved through engagement with the service providers and their leadership in the health and education sectors to plan for: (1) school hearing screening inclusion in budget allocations for financing the required human resources, equipment, and travel costs for policy implementation, (2) roles and expectations of the different sectors, (3) referral pathways, (4) programme monitoring and evaluation, and (5) considerations for a phased rollout.

Stakeholders in the current study demonstrated overwhelming support for implementation of school hearing screening in Botswana. This willingness by key government policymakers and service providers in the MoH and the MoESD was encouraging. The policymakers indicated that they would support the policy implementation processes through mobilisation of human and financial resources. The mobilisation of sufficient resources will be a key component for successful policy implementation.^40,49^ However, it is worrisome that the policymakers did not indicate where the funding to support implementation will be sourced from. The interviewed service providers from the MoH and MoESD recognised the need to have a dedicated budget for school hearing screening programmes from the government and for considering additional funding streams such as fundraising activities to bear the necessary operational costs.

Stakeholders from the health and education sectors acknowledged the current shortage of hearing healthcare professionals such as audiologists to conduct school hearing screening as a barrier. Some of the stakeholders suggested that the manpower shortage issue could be addressed by training available personnel, such as audiology assistants, community health workers, nurses, and teachers, to equip them with basic skills for conducting hearing screening and including them as part of the team involved in providing school hearing screening services. Other studies also reported shortage of skilled health personnel as one of the major barriers to the implementation of school hearing screening programmes,^50,51^ and suggested that using laypersons or personnel with minimal audiology training would reduce the human resources scarcity problem.^10,52^

School hearing screening services are usually an integral part of school health programmes.^53^ The availability of school health programmes in primary schools in Botswana presents an opportunity to integrate school hearing screening services into the existing school health programmes. Some of the service providers (nurses and teachers) suggested that upskilling members of their professions and integrating school hearing screening into their existing work roles would be a viable alternative for capitalising on the existing school health programmes. This multi-disciplinary integrated approach from the school health personnel could potentially promote sharing of the scarce resources such as human resource costs and logistics costs (e.g., transport), which may reduce the overall costs of the programme and minimise the learning interruptions in schools.^54^

The findings of the current study also suggested that there is a lack of collaboration among stakeholders from different government departments, such as the MoH and MoESD. The lack of stakeholder collaboration has been identified by other studies as one of the major barriers to policy implementation.^41,46,54^ The MoH policymakers and service providers in the present study highlighted that school health policy implementation could be enhanced by ensuring that there is role clarity and cohesion with adequate coordination and collaboration between the relevant stakeholders from the health, education, and other sectors. Previous studies have also reported that effective multi-sectoral stakeholder collaboration is a facilitator to successful implementation of hearing screening programmes for young children.^41,46^

### Limitations of the study

The present study did not include stakeholders from the Ministry of Local Government and Rural Development, even though this ministry is part of the tripartite custodians of the Botswana school health policy. Nevertheless, the findings of this study provide some valuable insights into understanding the knowledge and perspectives of some key government stakeholders from the health and education sectors concerning the provision of school entry hearing screening services.

## Conclusion

The present study demonstrates that policymakers were knowledgeable about the existence of the current national school health policy but had scant knowledge explicitly about a provision in the policy on school hearing screening. Most of the service providers from the health and education sectors were not aware of the existence of the current school health policy. However, there was universal support for policy implementation from the policymakers and service providers within the MoH and MoESD. These key government stakeholders were of the view that policy implementation was impeded by inadequate human and financial resources, lack of stakeholder collaborations, and lack of monitoring and evaluation. Policymakers and service providers underscored the importance of improving policy dissemination and awareness with respect to the school hearing screening provision to strengthen implementation uptake. The availability of school health programmes within the Botswana context presents a great opportunity for the integration of school entry hearing screening services into such programmes.

## References

[1] Mahomed-Asmail F. School-based hearing screening and diagnosis using automated and mobile health technologies [DPhil thesis] [homepage on the Internet]. Pretoria: University of Pretoria; 2015 [cited 2024 Oct 20]. Available from: http://hdl.handle.net/2263/57193

[2] Su JY, Guthridge S, He VY, Howard D, Leach, AJ. The impact of hearing impairment on early academic achievement in aboriginal children living in remote Australia: A data linkage study. BMC Public Health. 2020;20:1521. 10.1186/s12889-020-09620-6 33028291 PMC7542869

[3] Baltussen R, Li J, Wu LD, et al. Costs of screening children for hearing disorders and delivery of hearing aids in China. BMC Health Serv Res. 2009;9:64. 10.1186/1472-6963-9-64 19371419 PMC2679736

[4] Yong M, Liang J, Ballreich J, Lea J, Westerberg BD, Emmett SD. Cost-effectiveness of school hearing screening programs: A scoping review. Otolaryngol Head Neck Surg. 2020;162(6):826–838. 10.1177/0194599820913507 32228135

[5] Bamford J, Fortnum H, Bristow K, et al. Current practice, accuracy, effectiveness and cost-effectiveness of the school entry hearing screen. Health Technol Assess. 2007;11(32):1–168, iii–iv. 10.3310/hta11320 17683682

[6] Fortnum H, Ukoumunne OC, Hyde C, et al. A programme of studies including assessment of diagnostic accuracy of school hearing screening tests and a cost-effectiveness model of school entry hearing screening programmes. Health Technol Assess. 2016;20(36):1–178. 10.3310/hta20360 PMC4885008 27169435

[7] Banda FM, Powis KM, Mokoka AB, et al. Hearing impairment among children referred to a public audiology clinic in Gaborone, Botswana. Glob Pediatr Health. 2018;5:1–8. 10.1177/2333794X18770079 PMC5946350 29761140

[8] Khoza-Shangase K. Early hearing detection and intervention in South Africa: Exploring factors compromising service delivery as expressed by caregivers. Int J Pediatr Otorhinolaryngol. 2019;118:73–78. 10.1016/j.ijporl.2018.12.021 30590280

[9] Ansari MS. Hearing screening program for school going children in India: Necessity, justification, and suggested approaches. Egypt J Otolaryngol. 2021;37:118. 10.1186/s43163-021-00182-x

[10] Mahomed-Asmail F, Swanepoel D, Eikelboom RH, Myburgh HC, Hall J, III. Clinical validity of hearScreen™ smartphone hearing screening for schoolchildren. Ear Hear. 2016;37:e11–e17. 10.1097/AUD.0000000000000223 26372265

[11] Yong M, Panth N, McMahon CM, Thorne PR, Emmett SD. How the world’s children hear: A narrative review of school hearing screening programs globally. OTO Open. 2020;4(2):2473974X20923580. 10.1177/2473974X20923580 PMC7238315 32490329

[12] Ministry of Health. Botswana national school health policy and procedures manual. Gaborone: Government Printers; 1999.

[13] Taghizadeh S, Farhangi MA, Khodayari-Zarnaq R. Stakeholders’ perspectives of barriers and facilitators of childhood obesity prevention policies in Iran: A Delphi method study. BMC Public Health. 2021;21(1):2260. 10.1186/s12889-021-12282-7 34895191 PMC8665716

[14] Galárraga O, Bertozzi SM. Stakeholders’ opinions and expectations of the global fund and their potential economic implications. AIDS (London, England). 2008;22(Suppl. 1):S7–S15. 10.1097/01.aids.0000327618.01362.6a PMC2810424 18664957

[15] Kim H, Sefcik JS, Bradway C. Characteristics of qualitative descriptive studies: A systematic review. Res Nurs Health. 2017;40(1):23–42. 10.1002/nur.21768 27686751 PMC5225027

[16] Benoot C, Hannes K, Bilsen J. The use of purposeful sampling in a qualitative evidence synthesis: A worked example on sexual adjustment to a cancer trajectory. BMC Med Res Methodol. 2016;16(21):1–12. 10.1186/s12874-016-0114-6 26891718 PMC4757966

[17] Palinkas LA, Horwitz SM, Green CA, Wisdom JP, Duan N, Hoagwood K. Purposeful sampling for qualitative data collection and analysis in mixed method implementation research. Adm Policy Ment Health. 2015;42(5):533–544. 10.1007/s10488-013-0528-y 24193818 PMC4012002

[18] Guest G, Bunce A, Johnson L. How many interviews are enough?: An experiment with data saturation and variability. Field Methods. 2006;18:59–82. 10.1177/1525822X05279903

[19] Subedi KR. Determining the sample in qualitative research. Scholars J. 2021;4:1–13. 10.3126/scholars.v4i1.42457

[20] Vasileiou K, Barnett J, Thorpe S, Young T. Characterising and justifying sample size sufficiency in interview-based studies: Systematic analysis of qualitative health research over a 15-year period. BMC Med Res Methodol. 2018;18(1):148. 10.1186/s12874-018-0594-7 30463515 PMC6249736

[21] Bekele WB, Ago, FY. Sample size for interview in qualitative research in social sciences: A guide to novice researchers. REPAM. 2022;4(1):42–50. 10.46303/repam.2022.3

[22] Leon AC, Davis LL, Kraemer HC. The role and interpretation of pilot studies in clinical research. J Psychiatr Res. 2011;45(5):626–629. 10.1016/j.jpsychires.2010.10.008 21035130 PMC3081994

[23] Thabane L, Ma J, Chu R, et al. A tutorial on pilot studies: The what, why and how. BMC Med Res Methodol. 2010;10:1. 10.1186/1471-2288-10-1 20053272 PMC2824145

[24] Weller SC, Vickers B, Bernard HR, et al. Open-ended interview questions and saturation. PLoS One. 2018;13(6):e0198606. 10.1371/journal.pone.0198606 29924873 PMC6010234

[25] Busetto L, Wick W, Gumbinger C. How to use and assess qualitative research methods. Neurol Res Pract. 2020;2:14. 10.1186/s42466-020-00059-z 33324920 PMC7650082

[26] DeJonckheere M, Vaughn LM. Semistructured interviewing in primary care research: A balance of relationship and rigour. Fam Med Community Health. 2019;7(2):e000057. 10.1136/fmch-2018-000057 32148704 PMC6910737

[27] Taherdoost H. How to conduct an effective interview: A guide to interview design in research study. Int J Acad Res Manag. 2022;11(1):39–51.

[28] Naz N, Gulab F, Aslam M. Development of qualitative semi-structured interview guide for case study research. CSSRJ. 2022;3(2):42–52.

[29] Adeoye-Olatunde OA, Olenik NL. Research and scholarly methods: Semi-structured interviews. J Am Coll Clin Pharm. 2021;4(10):1358–1367. 10.1002/jac5.1441

[30] McGrath C, Palmgren PJ, Liljedahl M. Twelve tips for conducting qualitative research interviews. Med Teach. 2019;41(9):1002–1006. 10.1080/0142159X.2018.1497149 30261797

[31] Stacey K, Vincent J. Evaluation of an electronic interview with multimedia stimulus materials for gaining in-depth responses from professionals. Qual Res. 2011;11(5):605–624. 10.1177/1468794111413237

[32] Maurer K. Interview questions for qualitative research: An overview of providing advanced questions to interview participants. Sentio. 2024;6:38–54.

[33] QSR International Pty Ltd. NVivo 12 qualitative data analysis software. Denver, CO: Lumivero; 2018.

[34] Braun V, Clarke V. Using thematic analysis in psychology. Qual Res Psychol. 2006;3(2):77–101. 10.1191/1478088706qp063oa

[35] Lincoln YS, Guba EG. Naturalistic inquiry. Newbury Park, CA: SAGE Publications; 1985.

[36] Ghafouri R, Ofoghi S. Trustworth and rigor in qualitative research. Int J Adv Biotechnol Res. 2016;7(4):1914–1922.

[37] Anney VN. Ensuring the quality of the findings of qualitative research: Looking at trustworthiness criteria. JETERAPS. 2014;5(2):272–281.

[38] World Medical Association. World Medical Association Declaration of Helsinki: Ethical principles for medical research involving human subjects. J Am Med Assoc. 2013;310(20):2191–2194. 10.1001/jama.2013.281053 24141714

[39] Abdullahi M, Othman N. Bridging the gap between policy intent and implementation. J Sci Technol Innov Policy. 2020;6(1):1–10.

[40] Mthethwa RM. Critical dimensions for policy implementation. Afr J Public Aff. 2012;5(2):36–47.

[41] Shaibu S, Phaladze N. School health: The challenges to service delivery in Botswana. Prim Heath Care Res Dev. 2010;11(2):197–202. 10.1017/S1463423609990417

[42] Balane MA, Palafox B, Palileo-Villanueva LM, McKee M, Balabanova D. Enhancing the use of stakeholder analysis for policy implementation research: Towards a novel framing and operationalised measures. BMJ Glob Health. 2020;5(11):e002661. 10.1136/bmjgh-2020-002661 PMC7651378 33158851

[43] Das S, Seepana R, Bakshi SS. Perspectives of newborn hearing screening in resource-constrained settings. J. Otol. 2020;15(4):174–177. 10.1016/j.joto.2020.05.001 33293921 PMC7691834

[44] Alqudah O, Alqudah S, Al-Bashaireh AM, Alharbi N, Alqudah AM. Knowledge, attitude and management of hearing screening in children among family physicians in the Kingdom of Saudi Arabia. PLoS One. 2021;16(8):e0256647. 10.1371/journal.pone.0256647 34464417 PMC8407574

[45] Gracy D, Fabian A, Basch CH, et al. Missed opportunities: Do states require screening of children for health conditions that interfere with learning? PLoS One. 2018;13(1):e0190254. 10.1371/journal.pone.0190254 29342147 PMC5771574

[46] Naidoo N, Khan NB. Analysis of barriers and facilitators to early hearing detection and intervention in KwaZulu-Natal, South Africa. S Afr J Commun Disord. 2022;69(1):1–12. 10.4102/sajcd.v69i1.839 PMC8831925 35144437

[47] Baker EA, Brewer SK, Owens JS, Cook CR, Lyon AR. Dissemination science in school mental health: A framework for future research. Sch Ment Health. 2021;13(4):791–807. 10.1007/s12310-021-09446-6 PMC8053372 33897906

[48] Hudson B, Hunter D, Peckham S. Policy failure and the policy-implementation gap: Can policy support programs help? Policy Des Pract. 2019;2(1):1–14. 10.1080/25741292.2018.1540378

[49] Signé L. Policy implementation – A synthesis of the study of policy implementation and the causes of policy failure [homepage on the Internet]. Policy Paper, PP-17/03. Rabat: OCP Policy Center; 2017 [cited 2024 Nov 15]. Available from: http://www.ocppc.ma/sites/default/files/OCPPC-PP1703.pdf

[50] Maharjan M, Phuyal S, Shrestha M. Prevalence of hearing loss in school aged Nepalese children. Int J Pediatr Otorhinolaryngol. 2021;143:110658. 10.1016/j.ijporl.2021.110658 33636508

[51] Swanepoel D, Myburgh HC, Howe DM, Mahomed F, Eikelboom RH. Smartphone hearing screening with integrated quality control and data management. Int J Audiol. 2014;53(12):841–849. 10.3109/14992027.2014.920965 24998412

[52] Govender SM, Mars M. Assessing the efficacy of asynchronous telehealth-based hearing screening and diagnostic services using automated audiometry in a rural South African school. S Afr J Commun Disord. 2018;65(1):a582. 10.4102/sajcd.v65i1.582 PMC6111388 30035608

[53] Enekole O, Obukowho O, Ifeoma A. A critical appraisal of hearing impairment among primary schoolchildren in Port Harcourt, Nigeria. Int J Trop Dis Health. 2017;21(4):1–17. 10.9734/IJTDH/2017/31019

[54] Mohlabi DR, Van Aswegen EJ, Mokoena JD. Barriers to the successful implementation of school health services in the Mpumalanga and Gauteng provinces. S Afr Fam Pract. 2010;52(3):249–254. 10.1080/20786204.2010.10873983

